# The influence of climate variability on demographic rates of avian Afro-palearctic migrants

**DOI:** 10.1038/s41598-020-74658-w

**Published:** 2020-10-16

**Authors:** Tomáš Telenský, Petr Klvaňa, Miroslav Jelínek, Jaroslav Cepák, Jiří Reif

**Affiliations:** 1 Institute for Environmental Studies, Faculty of Science, Charles University, Prague, Benátská 2, 128 01 Praha 2, Czech Republic; 2 Institute of Vertebrate Biology, Academy of Sciences of the Czech Republic, Květná 8, 603 65 Brno, Czech Republic; 3 Bird Ringing Centre, National Museum, Prague, Hornoměcholupská 34, 102 00 Praha 10, Czech Republic; 4 Department of Zoology and Laboratory of Ornithology, Faculty of Science, Palacky University in Olomouc, 17. listopadu 50, 771 46 Olomouc, Czech Republic

**Keywords:** Ecology, Biodiversity, Population dynamics

## Abstract

Climate is an important driver of changes in animal population size, but its effect on the underlying demographic rates remains insufficiently understood. This is particularly true for avian long-distance migrants which are exposed to different climatic factors at different phases of their annual cycle. To fill this knowledge gap, we used data collected by a national-wide bird ringing scheme for eight migratory species wintering in sub-Saharan Africa and investigated the impact of climate variability on their breeding productivity and adult survival. While temperature at the breeding grounds could relate to the breeding productivity either positively (higher food availability in warmer springs) or negatively (food scarcity in warmer springs due to trophic mismatch), water availability at the non-breeding should limit the adult survival and the breeding productivity. Consistent with the prediction of the trophic mismatch hypothesis, we found that warmer springs at the breeding grounds were linked with lower breeding productivity, explaining 29% of temporal variance across all species. Higher water availability at the sub-Saharan non-breeding grounds was related to higher adult survival (18% temporal variance explained) but did not carry-over to breeding productivity. Our results show that climate variability at both breeding and non-breeding grounds shapes different demographic rates of long-distance migrants.

## Introduction

Climate is a key driver governing the spatial distribution of biodiversity^1,2^. Despite the widespread influence of climatic conditions on animal populations^3^, their impacts on species’ demographic rates are still insufficiently known in many organisms. This is particularly true for long-distance migratory birds. Study of climatic impacts on their demographic rates is challenging because these species travel for thousands of kilometres and visit different regions where they are exposed to various drivers^4–6^. Therefore, simultaneous assessments of climatic factors acting on demographic rates in different phases of migrants’ annual cycle are needed^7^. For this purpose, we focused on two important demographic rates reflecting the influence of climatic factors at the breeding and the non-breeding grounds—breeding productivity and adult survival, in Afro-palearctic migratory birds.

As each species is adapted to a specific range of climatic conditions^8^, climate change alters suitability of their habitats with consequences for their demography^9^. At the breeding grounds, migrants’ demography may be shaped by spring temperature acting upon breeding productivity^10,11^. Theoretically, two different relationships between temperature and breeding productivity are possible. In the first scenario, birds may benefit from higher temperatures due to earlier breeding enabling more breeding attempts per season resulting in higher number of juveniles produced per female^12^. In addition, higher temperatures result in higher amount of environmental energy, higher ecosystem productivity and thus more food resources available for birds during the breeding season^13^. The second scenario predicts the opposite pattern, i.e. a negative relationship between spring temperature and breeding productivity of long-distance migrants. Under this scenario, higher spring temperature results in altered interspecific interactions with the most influential trophic mismatch hypothesis in predator–prey interaction^14^. According to this hypothesis, warmer spring results in phenology shifts differing among trophic levels: since lower trophic levels typically shift faster^15,16^, a time lag between the peak of food supply for birds (typically insect larvae) and food demand of rearing nestlings occurs^17^. Consequently, nestlings have lower body condition resulting in reduced number of fledged juveniles and thus lower breeding productivity^18,19^.

The influence of spring temperature on birds’ breeding may be complex. While early spring temperature shapes the environment before or during the arrival of long-distance migrants and controls bud burst and invertebrate phenology^20^, temperature in late spring reflects conditions during breeding of long-distance migrants^3^ and may control the number of breeding attempts^21^. In addition, temperature may affect food availability^22^. It is thus interesting to study the relationships between the breeding productivity of long-distance migrants and spring advancement using different descriptors containing both early and late spring temperature, as well as variables describing ecosystem responses to weather conditions: growing degree days (accumulated temperatures above certain threshold) and dates of leaf unfolding^23–25^. One could expect that such variables describing the ecosystem responses will have stronger relationships to the breeding productivity than temperature per se.

At the Afrotropical non-breeding grounds, climatic factors shaping long-distance migrants’ demographic rates are mainly represented by the water availability in ecosystems^3^. The water availability may affect populations of long-distance migrants by its influence on adult survival and subsequent breeding productivity. Higher water availability increases both primary and secondary productivity, providing more food resources for birds^1^. Therefore, adult survival of Afro-palearctic migrants should be higher in years when more water is available in the ecosystems at their non-breeding grounds^26–28^. Climate variability at non-breeding grounds may also carry-over to affect the breeding performance^29^. For instance, a lower water availability in Africa may delay migrants’ arrival to breeding grounds^30,31^, reduce body condition after arrival^32^, and affect recruitment, brood size and number of fledglings^20,33^. However, non-breeding grounds of the Afro-palearctic migrants cover very large areas, ranging from highly arid Sahel zone to the humid rainforests in equatorial regions^34^. We can expect that the potential positive effect of higher water availability on adult survival and subsequent breeding productivity may be larger in regions with more severe water limitation^1^.

The majority of previous studies linking climate variability with demographic rates of long-distance migratory birds faced one or more of the following limitations: they focused on a single demographic parameter (e.g. productivity), did not consider both breeding and non-breeding grounds, studied a single species or even a single population, or covered only a few sampling sites (see^35^ for review). In this study, we aim to overcome these limitations by exploiting data collected for multiple species within a nation-wide citizen science programme based on constant effort long-term mist-netting of breeding birds. We assessed the influence of climate variability on migrants’ breeding productivity and adult survival. Specifically, we tested (1) whether the breeding productivity is higher in warmer or in colder springs; (2) whether the early spring or late spring temperature have stronger relationship to the breeding productivity; (3) whether the variables describing climate variability directly (i.e. temperatures) show stronger relationships to the breeding productivity than the measures related to resources (i.e. woody plant phenology) because we can expect that the endotherm organisms like birds will respond to the resources rather than to the temperatures per se; (4) whether adult survival and breeding productivity are positively related to higher water availability in sub-Saharan non-breeding areas.

## Results

### Breeding productivity

**Figure 1:**
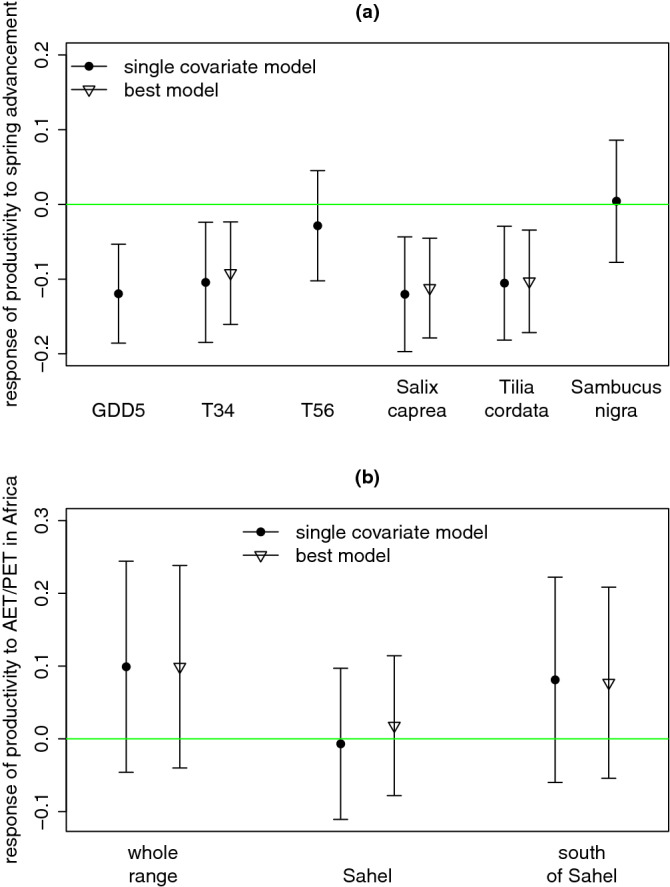
Responses (mean regression slopes ± 95% confidence intervals) of breeding productivity of long-distance migratory birds to (**a**) spring advancement at the breeding grounds and (**b**) water availability at the non-breeding grounds (carry-over effect). Results are shown for models with a single variable (single covariate model—filled circles) and for the best model combining one variable of spring advancement and one variable of carry-over effect (best model—empty triangles), if this latter model performed better than the single covariate model (see Table 1 and Supplementary Table S3 for full results). GDD5—growing degree days (accumulated temperature above 5°C); T34—mean temperature in March & April, T56—mean temperature in May & June; *Salix caprea, Tilia cordata, Sambucus nigra—*date anomaly of 10% leaf unfolding for given plant species (number of days before the long-term mean); AET/PET—ratio of actual to potential evapotranspiration in species’ whole sub-Saharan non-breeding range, its Sahelian part and the part south of the Sahel, respectively (see Supplementary Fig. S1b).

Considering all long-distance migrants (Supplementary Table S1) in a cross-species analysis, breeding productivity was negatively related to higher spring temperatures and earlier onset of leaf unfolding (Table 1a, Fig. 1a). Specifically, productivity was lower in years with higher early spring temperatures (i.e. in March and April), with higher GDD5 (growing degree days—accumulated temperature over 5°C, see Methods section for details), and with earlier leaf unfolding of *Salix caprea* and *Tilia cordata* (Fig. 1a). The proportion of explained variability ranged from 17 to 29%: it was higher for variables describing resource availability (i.e. leaf unfolding and GDD5) than for temperatures per se (Table 1a). Analysing data for individual species in separate models showed that the productivity was negatively related to at least one measure of spring advancement in five out of eight species (sedge warbler, marsh warbler, great reed warbler, willow warbler, and garden warbler), with the proportion of explained variability ranging from 39 to 84% (Supplementary Table S2).

**Table 1:** Relationships between the breeding productivity of long-distance migratory birds and variables describing (a) spring advancement at the breeding grounds; (b) water availability at the non-breeding grounds (indicating a so-called carry-over effect) and (c) at both breeding non-breeding grounds.

Explanatory variable	Model characteristics		Effect of spring advancement at the breeding grounds		Effect of water availability at the non-breeding grounds		Effect of population density	
	ΔAIC	R^2^_var^a^	slope (s. e.)	*p*-value	slope (s. e.)	*p*-value	slope (s. e.)	*p*-value
**a) Spring advancement at the breeding grounds**								
GDD5^b^	0.0	0.29	− **0.119 (0.034)**	** < 0.001**			− **0.164 (0.013)**	** < 0.001**
Early spring temperature^c^	5.0	0.17	− **0.104 (0.041)**	**0.011**			− **0.164 (0.013)**	** < 0.001**
Late spring temperature^d^	10.4	0.01	− 0.029 (0.038)	0.448			− **0.163 (0.013)**	** < 0.001**
*Salix caprea*^e^	2.5	0.27	− **0.120 (0.039)**	**0.002**			− **0.164 (0.013)**	** < 0.001**
*Tilia cordata*^e^	4.2	0.19	− **0.105 (0.039)**	**0.007**			− **0.164 (0.013)**	** < 0.001**
*Sambucus nigra*^e^	11.0	0.00	0.004 (0.042)	0.919			− **0.16 (0.013)**	** < 0.001**
**b) Water availability at the non-breeding grounds**								
AET/PET^f^ whole range^g^	9.2	0.01			0.099 (0.074)	0.184	− **0.164 (0.013)**	** < 0.001**
AET/PET Sahelian part^h^	11.0	0.00			− 0.007 (0.053)	0.892	− **0.163 (0.013)**	** < 0.001**
AET/PET south of Sahel^i^	9.7	0.01			0.081 (0.072)	0.255	− **0.164 (0.013)**	** < 0.001**
**c) Spring advancement at the breeding grounds and water availability at the non**-**breeding grounds**^j^								
GDD5^b^ + AET/PET whole range	0.1	0.29	− **0.121 (0.034)**	** < 0.001**	0.099 (0.071)	0.162	− **0.164 (0.013)**	** < 0.001**
GDD5^b^ + AET/PET south of Sahel	0.7	0.29	− **0.120 (0.034)**	** < 0.001**	0.077 (0.067)	0.250	− **0.164 (0.013)**	** < 0.001**
*Salix caprea* + AET/PET whole range	1.7	0.30	− **0.112 (0.034)**	**0.001**	0.120 (0.072)	0.097	− **0.165 (0.013)**	** < 0.001**
GDD5^b^ + AET/PET Sahelian part	1.9	0.29	− **0.123 (0.035)**	** < 0.001**	0.018 (0.049)	0.714	− **0.164 (0.013)**	** < 0.001**
*Tilia cordata* + AET/PET whole range	3.2	0.20	− **0.103 (0.035)**	**0.003**	0.126 (0.074)	0.087	− **0.165 (0.013)**	** < 0.001**
*Tilia cordata* + AET/PET south of Sahel	4.2	0.22	− **0.099 (0.034)**	**0.004**	0.099 (0.069)	0.150	− **0.165 (0.013)**	** < 0.001**
Early spring temp. + AET/PET whole range	4.8	0.17	− **0.092 (0.035)**	**0.008**	0.107 (0.072)	0.139	− **0.165 (0.013)**	** < 0.001**

Significant relationships are in bold.

^a^Proportion of temporal variance explained by explanatory variables after removing the variance explained by density dependence (see Methods section for details).

^b^Growing degree days—accumulated temperature above 5°C.

^c^Mean temperature in March and April.

^d^Mean temperature in May and June.

^e^Date anomaly of 10% leaf unfolding (number of days before the long-term mean).

^f^Ratio of actual to potential evapotranspiration in species’ sub-Saharan non-breeding ranges.

^g^Whole species’ sub-Saharan non-breeding ranges (see Fig. S1b).

^h^Sahelian part of species’ sub-Saharan non-breeding ranges (see Fig. S1b).

^i^South-of-Sahelian part of species’ sub-Saharan non-breeding ranges (see Fig. S1b).

^j^Ordered by ΔAIC; only models performing better than individual covariates are shown; all models can be found in Table S3.

The carry-over effect of water availability (AET/PET) in the sub-Saharan non-breeding grounds on long-distance migrants’ breeding productivity was weak (Table 1b,c). Although the regression slopes were mostly positive as expected, the relationships were not significant (Tables 1b,c, S3). Adding the sub-Saharan AET/PET as a second covariate along with the spring advancement brought minor improvement of model performance (lower AIC, higher explained variability—R^2^_var) only in a few models (Fig. 1b, Table 1b,c, Supplementary Table S3). Finally, breeding productivity was strongly negatively related to population density in both cross-species and species-specific models (Table 1, Supplementary Tables S2 and S3).

We repeated the analyses with short-distance migrants, partial migrants, and residents (Supplementary Table S1) because they may facilitate our understanding of the patterns found in long-distance migratory birds. We modified the cross-species models with the single spring advancement variable by adding the interaction between the spring advancement variable and migratory strategy (a categorical variable with three levels: long-distance migrants, short-distance migrants, residents/partial migrants). The pattern found for the long-distance migrants markedly contrasted with the relationships found for species of other migratory strategies (Supplementary Fig. S2). Whereas the long-distance migrants’ breeding productivity was negatively related to warmer springs in most of the relationships (Supplementary Table S4, Supplementary Fig. S2), the short distance migrants showed significantly positive responses to spring advancement variables, and residents and partial migrants non-significantly positive responses (Supplementary Fig. S2, Supplementary Table S4, S5).

### Adult survival

**Figure 2:**
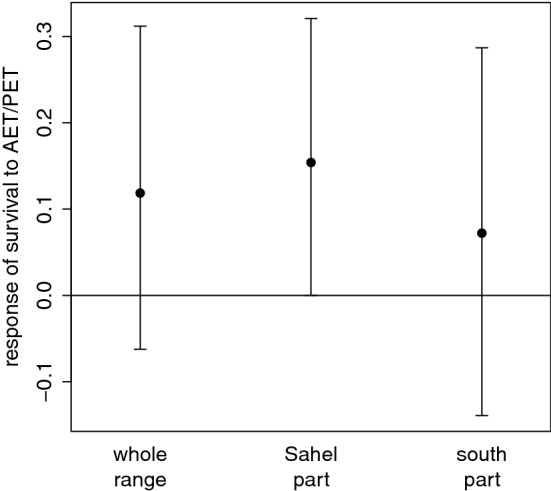
Responses (mean regression slopes ± 95% confidence intervals) of adult survival of long-distance migratory birds to water availability at their non-breeding grounds. Water availability is expressed as a ratio of actual to potential evapotranspiration (AET/PET) in (i) each species’ whole sub-Saharan non-breeding range, (ii) its Sahelian part and (iii) the part south of the Sahel (see Fig S1b). Each variable was tested in a single model.

When considering all long-distance migrants together in the cross-species models, adult survival was higher in years with higher AET/PET (i.e. higher water availability) in the Sahelian part of sub-Saharan non-breeding grounds (Fig. 2). In the species’ entire sub-Saharan non-breeding ranges, the relationship was also positive, but the confidence interval slightly overlapped zero, while in the species’ southern part of the sub-Saharan non-breeding grounds the relationship was non-significant (Fig. 2). Overall, AET/PET at non-breeding grounds explained from 11 to 18% (depending on the part of non-breeding ranges considered) of the variability in adult survival across all long-distance migrants (Table 2).

**Table 2:** Relationships between the adult survival of long-distance migrants and water availability (expressed as a ratio of actual to potential evapotranspiration, AET/PET) at the non-breeding grounds.

Explanatory variable	Slope	2.5%	97.5%	R^2^_dev^a^
AET/PET^b^ whole range^c^	0.119	− 0.062	0.312	0.153
AET/PET^b^ Sahelian part^d^	0.154	− 0.000	0.321	0.182
AET/PET^b^ south of Sahel^e^	0.072	− 0.139	0.287	0.114

^a^Proportion of deviance explained (see Methods section for details).

^b^Ratio of actual to potential evapotranspiration in species’ sub-Saharan non-breeding ranges.

^c^Whole species’ sub-Saharan non-breeding ranges (see Fig. S1b).

^d^Sahelian part of species’ sub-Saharan non-breeding ranges (see Fig. S1b).

^e^south-of-Sahelian part of species’ sub-Saharan non-breeding ranges (see Fig. S1b).

When performing the analysis for each species separately (Table 3), higher water availability in the Sahelian part of their non-breeding ranges was significantly positively related to adult survival for sedge warbler and common whitethroat. A significantly positive effect of higher water availability in the southern part of non-breeding ranges was found for reed warbler and willow warbler, and the adult survival of the latter species was also positively related to higher water availability in the whole non-breeding range (Table 3). The explained variability in these significant relationships ranged from 14 to 68% (Table 3).

**Table 3:** Species-level relationships between adult survival of long-distance migrants and water availability in non-breeding grounds estimated by a modified version of the Cormack-Jolly-Seber model.

Species	AET/PET (whole range)				AET/PET (Sahelian part)				AET/PET (south of the Sahel)			
	Slope	2.5%	97.5%	R^2^_dev^a^	Slope	2.5%	97.5%	R^2^_dev^a^	Slope	2.5%	97.5%	R^2^_dev^a^
*Acrocephalus arundinaceus*	− 0.124	− 0.783	0.535	0.009	− 0.002	− 0.556	0.551	< 0.001	− 0.162	− 0.868	0.544	0.014
*Acrocephalus palustris*	0.102	− 0.193	0.397	0.077	0.106	− 0.147	0.360	0.112	0.108	− 0.226	0.442	0.067
*Acrocephalus scirpaceus*	0.130	− 0.002	0.261	0.125	0.068	− 0.044	0.181	0.048	**0.146**	**0.008**	**0.283**	**0.143**
*Acrocephalus schoenobaenus*	0.123	− 0.020	0.265	0.332	**0.221**	**0.042**	**0.401**	**0.683**	0.078	− 0.069	0.225	0.125
*Phylloscopus trochilus*	**0.769**	**0.092**	**1.446**	**0.286**	0.571	− 0.106	1.247	0.194	**0.799**	**0.123**	**1.475**	**0.292**
*Sylvia borin*	− 0.253	− 0.591	0.086	0.201	− 0.016	− 0.369	0.337	0.001	− 0.282	− 0.610	0.047	0.270
*Sylvia communis*	0.334	− 0.150	0.818	0.150	**0.426**	**0.020**	**0.833**	**0.371**	0.037	− 0.462	0.537	0.002
*Sylvia curruca*	0.205	− 0.293	0.703	0.045	0.173	− 0.239	0.585	0.047	− 0.033	− 1.252	1.187	< 0.001

Water availability was expressed as a ratio of actual to potential evapotranspiration (AET/PET) in (i) each species’ whole sub-Saharan non-breeding range, (ii) its Sahelian part and (iii) the part south of the Sahel (see Fig S1b). Each variable was tested in a single model. Significant relationships (95% confidence limits not overlapping zero) are in bold. See Methods section for more details on model formulation and variable characteristics.

^a^Proportion of deviance explained (see Methods section for details).

## Discussion

Our study aimed to assess links between climate variability and two key demographic rates, productivity and adult survival, in avian long-distance migrants. We found that migrants’ breeding productivity was lower in warmer springs. Our models indicated that the early spring temperature performed better than the late spring temperature and that the temperature per se had lower explanatory power than the variables describing advancement of the ecosystems (i.e. growing degree days and date of woody plants’ leaf unfolding) explaining up to 29% of variability in the breeding productivity. In addition, the breeding productivity was negatively related to population density. Higher water availability at the sub-Saharan non-breeding grounds was positively related to migrants’ adult survival, explaining up to 18% of variability, and the positive effect of water availability was higher when considering the Sahel zone than when considering the southern part of the non-breeding grounds. In contrast, the carry-over effects of water availability at the non-breeding grounds on breeding productivity were weak.

Breeding productivity was negatively related to warmer springs, supporting the hypothesis that the more rapid spring advancement results in lower fitness of long-distance migrants, with population-level consequences^25,36,37^. When considering potential mechanisms, this result is consistent with the hypothesis of trophic mismatch^14^. It predicts that long-distance migrants suffer from fitness costs due to the spring advancement because they are unable to adjust timing of their breeding adequately^38^. Although the lower breeding productivity may be viewed as an obvious cost of warmer weather to long-distance migrants’ fitness, studies showing such a relationship are surprisingly infrequent. Negative relationship between productivity and spring temperature has been only seen in pied flycatcher in Poland and Spain^39–41^, barn swallow in Denmark^23^, seabirds^42–44^ and snow goose^17,45^ and Baird’s sandpiper^46^ in the Arctic. Many other studies on productivity show no such relationship^10,12,20,22,33,47^. This suggests that the warmer spring translates into population-level fitness costs only under certain circumstances and if these conditions are not met, no consequences of trophic mismatch are observed.

We suggest several ways how this may happen. Some bird species might be able to adjust the timing of their breeding to the more rapid spring advancement^48^. This is particularly the case for residents or short-distance migrants, species with more flexible phenology than the long-distance migrants^49^. Such species may even benefit from warmer springs, as our data imply for the short-distance migrants (Fig. S2, Tables S4, S5), possibly due to the prolongation of the breeding season^12^. Long-distance migrants though, due to the innate constraints of their annual cycle^50,51^, are not able to adjust their phenology as much as short-distance migrants and residents^50,52,53^. Nevertheless, they can adopt various different ways to buffer or escape the effect of mismatch: via density dependent juvenile survival^54^, diet flexibility^55^, adjusting the length of incubation^56^, and shortening the interval between arrival and breeding^57^. In addition, the effect of mismatch depends on the width and height of the prey abundance peak over season^14,58^. In the boreal and arctic zones, where cold climate strongly limits birds’ fitness^59^, breeding productivity will be higher in warmer springs irrespective to species’ migratory behaviour^11,60^. Warmer springs most likely provide more abundant food resources outweighing the negative effects of potential mismatch in the boreal conditions^58^. Taken together, we suggest that the failure to observe the effect of mismatch is not a flaw in the validity of its fundamental assumptions, but the consequence of the conditions prevailing in the study system.

It is important to realize that the negative relationship between the breeding productivity and spring advancement observed in our data may be also related to some other kinds of interspecific interactions unrelated to the trophic mismatch, namely interspecific competition and nest predation. In respect to competition, short-distance migrants and resident birds may benefit from warmer springs resulting in their more abundant local populations leading to the increased competitive pressure on long-distance migrants for common limiting resources^61,62^. However, the documented negative consequences of more intense interspecific competition between short-distance migrants or residents and long-distance migrants are restricted to the species breeding in nest holes^61,63,64^ which are indeed a key limiting resource in managed forests^65^. Yet, none of our focal long-distance migrants was a hole-nesting species. Concerning possible effects of the nest predation, it was found that birds may suffer from increased nest predation due to lower predators’ mortality in warmer conditions^66^. However, this factor acts across all species irrespective to their migratory strategies and it is not fully consistent with our results since short-distance migrants and residents did not show a negative relationship between breeding productivity and spring temperatures (Fig. S2, Tables S4,S5).

Breeding productivity was more tightly related to the early spring temperature (March, April) than to the late spring temperature (May, June). This result implies that breeding productivity is more affected by the weather conditions during or even before the species’ arrival to the breeding grounds than by the conditions the birds face during more advanced breeding phases. It may be caused by decisive impacts of the early spring phenology on food availability for birds in ecosystems^3^. However, the pattern may not hold for some other weather variables such as rainfall when heavy rains may cause serious breeding failure^67^, and thus reduce the breeding productivity. We also observed tighter relationships of the breeding productivity to the variables describing the environmental conditions, namely the GDD5 and the unfolding of *Salix caprea* and *Tilia cordata*, than to temperatures per se. It is likely that the leaf unfolding and GDD5 are biologically more relevant for endothermic organisms at higher trophic levels here represented by birds. While temperatures per se are not particularly important for such organisms^22^, the leaf unfolding and GDD5 show how the spring advancement is perceived by taxa at lower trophic levels the birds feed on. However, results for *Sambucus nigra* were less convincing. This discrepancy among the focal woody plant species might be caused by very high sensitivity of the latter shrub species to local temperatures^68^, leading to its extremely early onset of leaf unfolding in some years which is probably weakly associated with breeding phenology of birds.

The influence of climatic conditions at the non-breeding grounds on migrants’ survival has been discussed for decades^26,27^ and studies have tested their effects using single species^69^ or inferred their influence on migrants’ population dynamics without a specific test for relationships to adult survival^70^. However, broader formal assessments of the general validity of these relationships were lacking (but see^28^). Here we show that the influence of water availability at sub-Saharan non-breeding grounds was important when considering the northern (Sahelian) part, but less so in the southern part of the non-breeding ranges.

The key importance of moisture in the Sahel can be explained by the relatively low water availability and thus its stronger limiting effect on organisms in the northern part of sub-Saharan Africa compared to more southern regions^71^. In addition, trans-Saharan migrants frequently stopover in the Sahel to restore the reserves after and before crossing the Sahara Desert^72^. Drought events in the Sahel thus have serious consequences even for species spending the winter further south^72^. However, in our study the importance of different non-breeding regions differed at the species level. Together with previous studies, we found that conditions in the Sahel were important for sedge warbler and common whitethroat^26,27^, whereas willow warbler and reed warbler were more affected by water availability in southern parts of their non-breeding ranges.

In contrast to the importance of the non-breeding grounds’ climate variability for adult survival, we did not detect any significant carry-over effects of the water availability at the non-breeding grounds on the breeding productivity. Previous studies also reported a weak carry-over effect (e.g.^73^.). It can be explained by rather indirect causal pathways that act in these relationships when other factors affecting the breeding productivity can balance the adverse effects of water stress at the non-breeding grounds. For example, limited water availability results in increased migrant mortality during winter, but the individuals which do arrive successfully to the breeding grounds enjoy weaker intraspecific competition^74^. In turn, their body condition can improve and no adverse consequences on breeding performance may be observed. Indeed, the importance of intraspecific competition for breeding performance has been frequently reported (e.g.^73^.).

In conclusion, we found that climate variability (i.e. spring advancement at the breeding grounds and water availability at the non-breeding grounds) affected both the breeding productivity and adult survival of long-distance migratory birds. Regression slopes and explained variability show that the relationships between spring advancement at the breeding grounds and the breeding productivity were stronger than the relationships between water availability at the non-breeding grounds and adult survival. However, it is difficult to correctly compare the importance of conditions at breeding and non-breeding grounds. As there are great uncertainties in locations of species’ non-breeding grounds^6,75^, we cannot exclude that the suggested higher importance of the breeding ground conditions is only observed due to more precise data collected at the breeding grounds: while the breeding productivity is related to the conditions recorded in relatively close vicinity of the sampling sites, the adult survival is related to the climatic data covering the areas of several orders of magnitude larger where the individuals can search for suitable conditions. This may result in inevitably weaker relationships. Therefore, to make clear judgements about the importance of the breeding vs. non-breeding ground conditions, we would need much more precise location of the non-breeding grounds for the studied populations of the focal species. Recent advancement of modern tracking techniques indicates that this might be achieved in near future^76^. Subsequent studies may link demographic rates we study here with species’ population dynamics or study gradients in demographic rates across the species’ climatic ranges at continental scales.

## Methods

### Avian data

Bird population data were collected within the Constant Effort Sites (CES) mist-netting scheme in the Czech Republic during 2004–2014. The CES scheme focuses on the annual collection of capture-mark-recapture data for birds using a network of skilled volunteers using a standard protocol^77^. For the present study, we used data from a total of 42 sites scattered throughout the country. At each site, birds were mist-netted during 9 visits in ca. 10-day intervals covering the advanced breeding season (May–July). We used data on all species available, except for species with less than 30 inter-annual recaptures and species recorded at less than 7 sites in any of the years, as these captures could not be used to get reliable estimates of demographic rates. In the end, we used data on eight long-distance migratory species (Supplementary Table S1). All of them are small passerines breeding in reed and scrub habitats and laying a single brood per season in the Czech Republic^78^.

To identify the non-breeding grounds of the focal species, we used data on species’ non-breeding ranges provided by BirdLife International (https://datazone.birdlife.org/species/search). To identify non-breeding grounds of Czech long-distance migrant populations, we intersected these ranges with those of^77^. In^77^, the ranges were defined based on all ringing recoveries collected in the Czech Republic (from the start of bird ringing in 1920s to 2002, with updates till 2008). Because the number of ringing recoveries from sub-Saharan Africa was too low for the exact location of the migrants’ non-breeding grounds, the authors of^77^ divided sub-Saharan Africa into four large geographic regions and matched the non-breeding range of each species with one or more of these four regions (Supplementary Fig. S1a). By that means, we obtained the best information on locations of non-breeding ranges for Czech populations of the focal species available (Supplementary Fig. S1b). Using a global map of ecoregions^79^ (http://ecoregions2017.appspot.com/), we further split the species’ non-breeding ranges into a Sahelian part and a part south of the Sahel (Supplementary Fig. S1b) considering three ecoregions as those covering the Sahel zone: Sahelian Acacia savanna, Djibouti Xeric shrublands and Horn of Africa Xeric shrublands.

### Climate variables

To describe spring advancement at the breeding grounds, we used the following variables provided on an annual basis from 2004 to 2014 by the Czech Hydrometeorological Institute: mean temperature in early spring (March and April), which shapes environmental conditions preceding or during the arrival of long-distance migrants and controls bud burst and invertebrate phenology^20^; mean temperature in late spring (May and June), which reflects conditions during the breeding of long-distance migrants; growing degree-days in March and April (GDD5—here, the sum of mean daily temperatures above 5°C since the first day of spring onset, which is defined as the first day of the first period of six consecutive days with mean temperature ≥ 5°C since 1st March); date anomaly of 10% leaf unfolding of three tree species (*Salix caprea*, *Tilia cordata* and *Sambucus nigra*)—these woody plant species reflect in a wide range of ecological conditions (from humid to dry habitats and from lowlands to highlands) and they are well-represented in sites where the focal bird species breed. The phenophase of 10% unfolding means that 10% of the plant leaves have started to unfold, having the whole midrib visible already, but the leaf is still partially folded^80^. We expressed the date anomaly of 10% leaf unfolding as a number of days before the long-term mean to reconcile the direction of all variables describing spring advancement (higher values—earlier spring onset) facilitating comparison of their performance. Temperatures and GDD5 were calculated as the average from 18 meteorological stations situated in proximity to the monitored area; the 10% leaf unfolding date anomaly was calculated as the median date from all 26–27 meteorological stations across the Czech Republic where these data were available (see https://portal.chmi.cz/files/portal/docs/poboc/OS/stanice/ShowStations_CZ.html).

To measure water availability at non-breeding grounds in sub-Saharan Africa, we used the ratio of actual to potential evapotranspiration (AET/PET). Unlike commonly used measures like rainfall, which is a measure of water income, or indirect measures like NDVI, AET/PET much more directly quantifies the amount of water available in the ecosystem^81^ and is reported to be better predictor of bird distribution^82^. The monthly data on AET/PET were obtained from MODIS MOD 16^83^, in particular MOD16A2 monthly data in the GeoTIFF raster format with 0.5 degree resolution. We considered three variants of non-breeding ranges (Fig. S1b): (1) the whole non-breeding range, (2) the Sahelian part and (3) the southern part (see above). For each species and range variant, we extracted the average AET/PET for each month. Then we averaged the monthly values across each winter season (September to April). The months included in the winter season were selected based on published information on the occurrence of Czech populations of the focal migratory species in sub-Saharan Africa^77,84,85^.

### Statistical analyses

#### Breeding productivity

To relate the breeding productivity to the spring conditions at the breeding grounds and to the carry-over effect of water availability (AET/PET) in the sub-Saharan non-breeding grounds, we employed logistic regression using generalised linear mixed models (GLMM), using the glmer function in ‘lme4’ package^86^ in R^87^. We fitted species-level models (separate model for every species), as well as cross-species models (see Table 4 for a summary of all fixed and random effects in these models). Response variable in the logistic regression was the total number of juveniles (supplied as “successes”) and adults (supplied as “failures”) caught at a given site and year, thus representing the ratio of juveniles to adults. Since our intention was also to compare different variables for both spring advancement and water availability in Africa, we considered three variants of models with different combinations of explanatory variables: (1) single spring advancement variable, (2) single AET/PET variable, (3) single spring advancement and AET/PET variable. Since taking into account the confounding effect of density dependence is crucial in analysis of the breeding productivity^11^, we also included it in the model. We defined density as a temporal anomaly of total number of adults of given species at a given site—i.e. the number of adults normalized per species and site. Since the sites effectively create pseudoreplicates of the time-series (responses are more similar within a given year), and also to account for the breeding productivity difference between sites, we included random effects ‘species:year’ and ‘species:site’, respectively (year and site in species-level models). We also included a random intercept effect for species in cross-species models. It would have been beneficial to include a random slope effects of the covariates as well, but some of those models resulted in singular fit, suggesting they were too complex, so we did not use it in order to keep models comparable. We only used the random slope effect to evaluate the proportion of temporal variance explained by climatic covariates (R^2^_var, see below) because the explanatory power of these covariates would be underestimated without the species-specific component of the slope. To calculate the proportion of explained temporal variance (R^2^_var), we adopted the method proposed in^88^, their chapter 5.7, and in^89^, their p. 378, Eq. 7, generalized to cross-species model: R^2^_var was computed as ($(\sigma^{2}_{{{\text{total}}}} - \sigma^{2} )/\sigma^{2}_{{{\text{total}}}}$, where $\sigma^{2}$ is the residual temporal variance, i.e. the variance of the species:year random effect, and $\sigma^{2}_{{{\text{total}}}}$ is the total temporal variance (after the effect of density dependence is removed), i.e. the variance of the species:year random effect of the corresponding model with climatic covariates removed (but still including the density dependence).

**Table 4:** Fixed and random effects structure of the generalized linear mixed models relating breeding productivity of long-distance migratory birds to climate variability.

Fixed effects	Random effects
**Cross-species models**^a^	
cov_spring + cov_Africa + density	(1|Species:Year) + (1|Species) + (1|Species:Site)
cov_spring + cov_Africa + density	(1|Species:Year) + (1 + cov_spring + cov_Africa|Species) + (1|Species:Site)
**Species-level models**	
cov_spring + cov_Africa + density	(1|Year) + (1|Site)

Cross-species models contained all species of long-distance migrants together, whereas each species-level model contained a single species. “cov_spring” is a variable measuring spring advancement at the breeding grounds, “cov_africa” is a variable measuring water availability (expressed as a ratio of actual to potential evapotranspiration) at the non-breeding grounds. Some models used only one of these two covariates (see Methods section for more details). Random effects use common notation used in R language (“:” denotes interaction, “1” before vertical bar “|” denotes random intercept effect; covariate before “|” denotes random slope effect).

^a^Model without and with random slope effect, respectively.

In the productivity analysis, we excluded the year 2013 because of extreme weather conditions during late spring, with abnormally heavy rain and floods in May and June resulting in extraordinary low productivity clearly unrelated to our focal effect of spring advancement.

#### Adult survival

To assess the impact of water availability in sub-Saharan Africa on the survival of adult birds of each long-distance migrant species, we used the Cormack–Jolly–Seber (CJS) survival model. Inter-annual survival between two consecutive years was modelled as a linear function (after logit transformation) of AET/PET in a given non-breeding season (September-April, see section “Climate variables” above) in the non-breeding range of a given species. Similarly to the modelling of carry-over effects, we considered three variants of non-breeding areas (see section “Avian data” above) in separate models.

Estimates of adult survival are often biased due to the presence of transient individuals, i.e. those being captured, but not being residents at a site, in the focal population^90^. We accounted for the presence of transients using the method described by^28^, which is an implementation of so-called Pradel’s model^90^ using extended capture histories. In this method, an individual is considered as a resident (i.e. non-transient) if captured in two different occasions (regardless if they occur in the same breeding season or not). Transience, i.e. the probability that an individual is transient, was modelled as constant. We tested the temporal trend in transience by modelling it as a linear function of year, but it was not significant for any of the species. Models were computed in the program MARK ran within the R-package ‘RMark’^91^.

Next, we computed the percentage of deviance explained by the climate variable (R^2^_dev) using the ANODEV method^88^ (their p. 378, Eq. 6). Then, to assess the overall impact across species, we computed the mean of all AET/PET coefficients across all species, taking also their standard errors into account using the meta-analytic Bayesian approach described in^88^. This model was fit in JAGS, with 3 chains, 200 000 iterations and disposing the first half as burn-in.

#### Additional information on modelling techniques

All Bayesian models were fitted with uninformative priors. Convergence was tested using a potential scale reduction factor^92^ using the gelman.diag function from the R package ‘coda’^93^. Since we were interested in responses of present-day species to environmental changes within a time period considerably shorter than the time scales of evolutionary processes, we did not control for shared ancestry in cross-species analyses. These approaches would be appropriate for analyses investigating variability in heritable traits affected by common evolutionary history of the focal species, which is not the case for demographic rates (see^94,95^ for more arguments on this topic). All explanatory variables describing spring advancement at the breeding grounds and water availability at the non-breeding grounds were standardized to zero mean and unit variance before the analysis.

## Ethics statement

CES programme is organized by the Czech Bird Ringing Centre of the National Museum, Prague, with all voluntary ringers being attested by the Czech Bird Ringing Centre to conform the regulation no. 152/2006 of the Ministry of Environment of the Czech Republic on The Exception of Bird Capturing for Ringing Purposes based on the law no. 114/1992 on The Nature and Landscape Protection. The experimental protocol of CES was approved by the National Museum, Prague. The study was performed in accordance with relevant guidelines and regulations.

## Supplementary information

Supplementary Information.

Supplementary Table S2.

Supplementary Table S3.

Supplementary Table S4.

Supplementary Table S5.

## Data Availability

Data on water availability extracted from free online resources and all bird data are available in Dryad repository^96^. Data collected by the Czech Hydrometeorological Institute and used in this study cannot be further distributed by the authors, but these data can be provided by this institute upon request.
