# Supplementary material for: The influence of climate variability on demographic rates of avian Afro-palearctic migrants
Source: Sci Rep. 2020 Oct 16;10:17592. doi: 10.1038/s41598-020-74658-w (PMC7567877; doi:10.1038/s41598-020-74658-w)
Supplement: Supplementary file 1 — Supplementary Information. [file 41598_2020_74658_MOESM1_ESM.docx]

**Supplementary online materials**

**The influence of climate variability on demographic rates of avian Afro-palearctic migrants**

Tomáš Telenský^1,2^, Petr Klvaňa^3^, Miroslav Jelínek^3^, Jaroslav Cepák^3^, Jiří Reif^1,4^*

^1^Institute for Environmental Studies, Faculty of Science, Charles University, Prague, Benátská 2, 128 01 Praha 2, Czech Republic

^2^Institute of Vertebrate Biology, Academy of Sciences of the Czech Republic, Květná 8, 603 65 Brno, Czech Republic

^3^Bird Ringing Centre, National Museum, Hornoměcholupská 34, 102 00 Praha 10, Czech Republic

^4^Department of Zoology and Laboratory of Ornithology, Faculty of Science, Palacky University in Olomouc, 17. listopadu 50, 771 46 Olomouc, Czech Republic

**Correspondence author:** Jiri Reif. Address: Faculty of Science, Charles University, Prague, Benátská 2, 128 01 Praha 2, Czech Republic. Email: jirireif@natur.cuni.cz

**Supplementary Fig. S1:** (a) Division of sub-Saharan Africa into four geographic regions used for delineation of non-breeding ranges (W – West Africa, E – East Africa, S – South Africa, C – Central Africa) according to [1]; (b) sub-Saharan non-breeding ranges of Czech populations of the focal long-distance migrants. Yellow color denotes the area belonging to the Sahelian ecoregion according to [2], see https://ecoregions2017.appspot.com/, blue color denotes the southern part of the non-breeding range. For more details on range delineation, see the Methods section “Avian data”. The maps were created (a) in QGIS 3.6.1 [3] using data from [1], and (b) in R [4] using data from [1,2,5] for (b).

a)


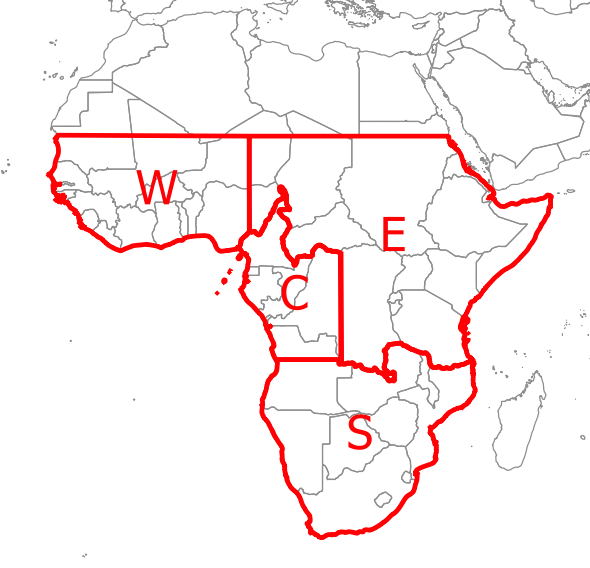


b)

**Supplementary Table S1:** Characteristics of the species involved in this study: migratory strategy (LD - long-distance migrant, SD - short-distance migrant, PAR - partial migrant, RES - resident), total number of adult individuals captured and total number of individuals captured in two different years (inter-annual recaptures).

| Species | Common name | Migratory strategy | Total captured | Inter-annual recaptures |
| --- | --- | --- | --- | --- |
| *Acrocephalus arundinaceus* | Great Reed Warbler | LD | 703 | 31 |
| *Acrocephalus palustris* | Marsh Warbler | LD | 2681 | 121 |
| *Acrocephalus scirpaceus* | Reed Warbler | LD | 8730 | 712 |
| *Acrocephalus schoenobaenus* | Sedge Warbler | LD | 3425 | 259 |
| *Emberiza citrinella* | Yellowhammer | RES | 776 | 64 |
| *Emberiza schoeniclus* | Reed Bunting | SD | 1575 | 94 |
| *Parus caeruleus* | Blue Tit | PAR | 1128 | 92 |
| *Parus major* | Great Tit | PAR | 914 | 73 |
| *Phylloscopus collybita* | Chiffchaff | SD | 1797 | 104 |
| *Phylloscopus trochilus* | Willow Warbler | LD | 614 | 40 |
| *Prunella modularis* | Dunnock | SD | 469 | 56 |
| *Sylvia atricapilla* | Blackcap | SD | 3504 | 202 |
| *Sylvia borin* | Garden Warbler | LD | 1033 | 92 |
| *Sylvia communis* | Common Whitethroat | LD | 818 | 76 |
| *Sylvia curruca* | Lesser Whitethroat | LD | 486 | 60 |
| *Turdus merula* | Eurasian Blackbird | PAR | 961 | 96 |

**Supplementary Table S2:** Relationships between breeding productivity of particular long-distance migratory bird species and variables reflecting spring advancement at the breeding grounds and water availability at the non-breeding grounds. Each table row corresponds to one single-species model, featuring (i) spring advancement at the breeding grounds; (ii) water availability at the non-breeding grounds (indicating a so-called carry-over effect) and (iii) both spring advancement and the carry-over effect. Significant relationships are in bold. See the Methods section for more details on model formulation and variable characteristics.

SEE THE EXCEL FILE

**Supplementary Table S3**: Relationships between breeding productivity of long-distance migrants and climate variability. Each table row corresponds to one cross-species model, featuring (i) spring advancement phenology at the breeding grounds; (ii) water availability at the non-breeding grounds (indicating a so-called carry-over effect) and (iii) both spring advancement phenology and the carry-over effect. The effect of population density was taken into account. Significant relationships are in bold. See the Methods section for more details on model formulations and variable characteristics.

SEE THE EXCEL FILE

**Supplementary** **Fig S2:** Responses (mean regression slopes ±95% confidence intervals) of breeding productivity of species with different migratory strategies (i.e. long-distance migrants, short-distance migrants, residents & partial migrants) to spring advancement at the breeding grounds. Each variable was tested in a separate model as a single covariate (see Table S4 for full results). GDD5 – growing degree days (accumulated temperature above 5°C, black squares); T34 – mean temperature in March & April (black dots); T56 – mean temperature in May & June (black triangles); *Salix caprea* (black diamonds)*, Tilia cordata* (empty diamonds)*, Sambucus nigra* (empty triangles) – date anomaly of 10% leaf unfolding for given plant species (number of days before the long-term mean).


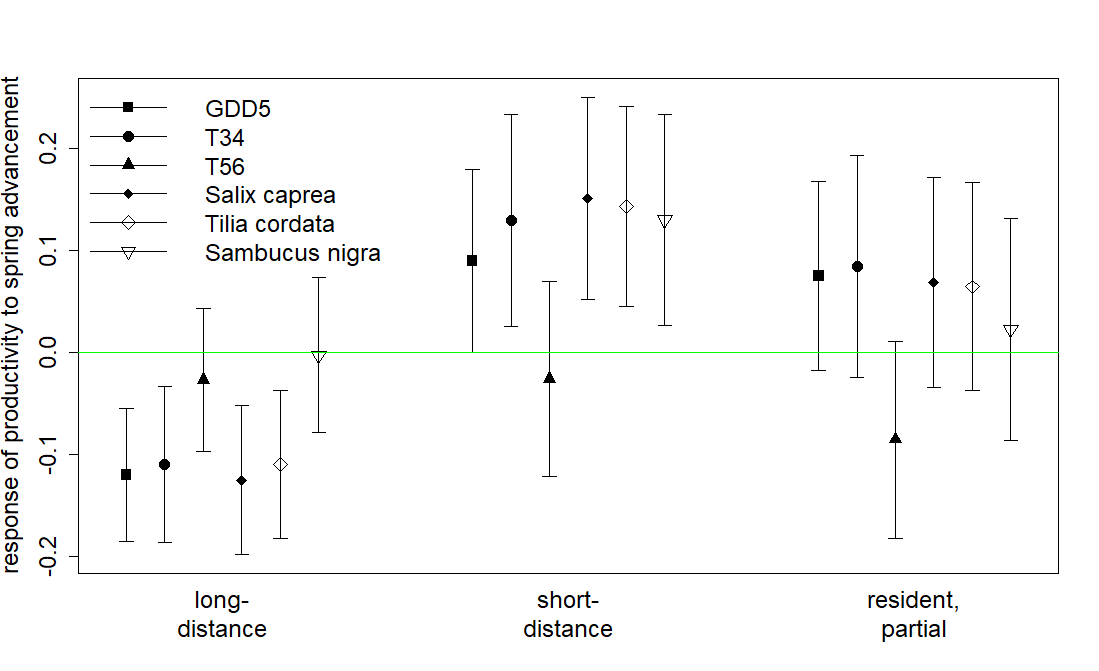


**Supplementary** **Table S4:** Relationships between breeding productivity of bird groups defined by different migratory strategies and spring advancement at the breeding grounds. Each variable was tested in a single cross-species model taking also the potential effect of population density (estimated across all bird groups together) into account. Significant relationships (95% confidence limits not overlapping zero) are in bold. See the Methods section for more details on model formulation and variable characteristics.

SEE THE EXCEL FILE

**Supplementary** **Table S5:** Relationships between breeding productivity of particular species of short-distance migrants (SD), partial migrants (PAR) and residents (RES) and spring advancement at the breeding grounds. Each table row corresponds to one single-species model. Significant relationships are in bold. See the Methods section for more details on model formulation and variable characteristics

SEE THE EXCEL FILE

**References**

[1] Cepák, J. *et al.* (eds) *Czech and Slovak Bird Migration Atlas* (Aventinum, 2008).

[2] Dinerstein, E. *et al.* An ecoregion-based approach to protecting half the terrestrial realm. *BioScience* **67**, 534-545 (2017).

[3] QGIS Development Team: QGIS Geographic Information System. Open Source Geospatial Foundation Project. https://qgis.osgeo.org (2019).

[4] R Core Team. R: A language and environment for statistical computing. http://www.r-project.org/ (2016).

[5] BirdLife International: http://datazone.birdlife.org/species/search (2019).
